# Effects of Flavourzyme and Alkaline Protease Treatment on Structure and Allergenicity of Peanut Allergen Ara h 1

**DOI:** 10.17113/ftb.62.01.24.8064

**Published:** 2024-03

**Authors:** Erlian Shu, Shuo Wang, Xiangxiang Kong, Xiaodong Sun, Qiaoling Yang, Qin Chen, Bing Niu

**Affiliations:** 1 School of Life Sciences, Shanghai University, No. 99, Shangda Road, Baoshan District, 200444 Shanghai, PR China; 2 School of Medicine, Shanghai University, No. 99, Shangda Road, Baoshan District, 200444 Shanghai, PR China; 3 School of Environmental and Chemical Engineering, Shanghai University, No. 99, Shangda Road, Baoshan District, 200444 Shanghai, PR China

**Keywords:** protease treatment, protein structure analysis, Ara h 1, peanut allergen

## Abstract

**Research background:**

Peanut allergy poses a significant threat to human health due to the increased risk of long-term morbidity at low doses. Modifying protein structure to affect sensitization is a popular topic.

**Experimental approach:**

In this study, the purified peanut allergen Ara h 1 was enzymatically hydrolysed using Flavourzyme, alkaline protease or a combination of both. The binding ability of Ara h 1 to antibodies, gene expression and secretion levels of the proinflammatory factors interleukin-5 and interleukin-6 in Caco-2 cells was measured. Changes in the secondary and tertiary structures before and after treatment with Ara h 1 were analysed by circular dichroism and sodium dodecyl sulfate-polyacrylamide gel electrophoresis (SDS-PAGE).

**Results and conclusions:**

The results indicated a decrease of the allergenicity and proinflammatory ability of Ara h 1. The evaluation showed that the Flavourzyme and alkaline protease treatments caused particle shortening and aggregation. The fluorescence emission peak increased by 3.4-fold after the combined treatment with both proteases. Additionally, the secondary structure underwent changes and the hydrophobicity also increased 8.95-fold after the combined treatment.

**Novelty and scientific contribution:**

These findings partially uncover the mechanism of peanut sensitization and provide an effective theoretical basis for the development of a new method of peanut desensitization.

## INTRODUCTION

Peanuts are rich in fat and protein and contain vitamins, calcium, iron and other minerals that are beneficial to health (*1*, *2*). They are popular in the processed food industry because of their delightful taste and nutritional value. Peanut allergy symptoms include lip and face oedema, tracheal stenosis, urticaria, twitching, vomiting, diarrhoea, asthma, anaphylactic shock, among others. They can cause severe discomfort and even death (*3*). Peanut allergies are caused by 17 allergens collectively known as Ara h 1 to Ara h 17 (*4*). Notably, Ara h 1-3 and Ara h 6 are recognised by 90 % of the sera of allergic patients (*5*). Ara h 1 is the most abundant allergen in peanuts, accounting for 12-16 % of the total peanut protein. It has been reported that serum-specific IgE for Ara h 1 is present in 75 % of individuals allergic to peanuts (*6*). Chosen for its abundance and high specificity, Ara h 1 is currently under investigation for methods to mitigate its allergenicity, with existing research in this area being inconclusive.

Peanut allergy is a severe food allergy, which manifests as an IgE-mediated immune response triggered by the consumption of peanuts or peanut-containing products (*7*, *8*). Protease treatment, commonly employed in food processing, is a key strategy for reducing the allergenicity of peanut proteins (*9*-*11*). Protein structures undergo changes during cross-linking and aggregation (*12*), either disrupting the original protein epitope or generating new ones, thereby affecting the allergenicity of peanut proteins. Cabanillas *et al*. (*13*) reported a 65 % inhibition of peanut protein and IgE binding after Flavourzyme treatment. In another study, Yu and Mikiashvili (*14*) processed peanuts with alkaline protease, papain, neutral protease and bromelain, and observed different hydrolytic effects on specific allergens. Among these, alkaline proteases were most effective in reducing the concentration and allergenicity of raw peanut allergens. Flavourzyme, alkaline proteases, exoproteases and endoproteases substantially reduced protein allergenicity. After treatment with pepsin and trypsin, allergenic peanut proteins were degraded into small peptides (*15*). In this study, Ara h 1 was hydrolysed with two harmless edible enzymes, Flavourzyme and alkaline protease, which were used individually and combined. Studies suggest that hydrolysing peanut allergenic proteins with non-specific protease can significantly reduce peanut allergenicity (*16*). However, the mechanism of protease treatment on peanut allergenicity is yet to be studied.

Roth-Walter *et al*. (*17*) used Caco-2 cells as a model to study the allergenicity of milk protein, and the results show that the aggregation of soluble milk β-lactoglobulin can induce the production of IgE antibodies and cytokines in Caco-2 cells, such as IL-6, IL-8, IL-15 and thymic stromal lymphopoietin (TSLP). Previous studies have shown that the inflammatory factors IL-5 and IL-6 play important roles in peanut allergic reactions, which are induced by the Th2 response once susceptible individuals consume peanuts (*18*). Basophils can be activated and recruited by these factors to release their cellular contents, which trigger tissue damage and inflammation in the body (*19*). Therefore, IL-5 and IL-6 are used as inflammatory biomarkers to evaluate allergenicity in the Caco-2 cell model.

In this study, we investigated the structural changes in Ara h 1 and its ability to bind to antibodies following hydrolysis with proteases (Flavourzyme, alkaline protease, or their combination). In addition, we investigated changes in IL-5 and IL-6 gene expression and secretion, which indicated the effects of protease treatment on Ara h 1 structure and allergenicity in Caco-2 cells. This study provides a basis for understanding the mechanism of peanut allergy and developing new desensitization methods.

## MATERIALS AND METHODS

### Purification of Ara h 1

Crude peanut protein was extracted and Ara h 1 purified as previously described by Wang *et al.* (*2*). Peanuts were purchased from Qiannuo (Shanghai, PR China). Ara h 1 was purified using a protein purification system (AKTA pure; General Electric, Boston, MA, USA) and column (HiTrap Q HP; General Electric) through anion exchange chromatography.

### Enzymatic hydrolysis of Ara h 1

The alkaline protease and Flavourzyme were purchased from Macklin Biochemical Technology Co., Ltd. (Shanghai, PR China). The hydrolysis conditions for Flavourzyme were as follows: the protease to protein amount of substance ratio of 10:100, reaction at 60 °C in a water bath for 100 min and then at 100 °C for 10 min to inactivate the protease. The hydrolysis conditions of alkaline protease were as follows: the amount of substance ratio of protease to protein 4:100, water bath at 55 °C for 100 min, and then at 100 °C for 10 min to inactivate the protease. The hydrolysis conditions of the two proteases were as follows: the amount of substance ratio of Flavourzyme to protein 5:100, water bath at 60 °C for 100 min, then at 100 °C for 10 min to inactivate the protease. After that, 1 mL of 2 % alkaline protease was added in a water bath at 55 °C for 100 min and then at 100 °C for 10 min to inactivate the protease.

### SDS-PAGE

Proteins were separated using a PAGE Gel Fast Preparation Kit (Epizyme, Shanghai, PR China). The experimental procedures were described by Wang *et al.* (*2*). We used a gel with a thickness of 1.0 mm for electrophoresis. An upper gel voltage of 80 V was applied for 20 min and a lower gel voltage of 120 V was applied for 60 min. The gel was stained with Coomassie bright blue R-250 (Solarbio, Shanghai, PR China) for 30 min, decolourized overnight for 12 h and the electrophoretic image of the protein was obtained.

### Observation by atomic force microscope

An atomic force microscope (AFM 5500; Agilent Technologies, Santa Clara, CA, USA) was used to observe the surface morphology of Ara h 1. A volume of 10 μL of Ara h 1 (0.1 mg/mL) solution was dropped on the surface of the sample carrier. After resting for 10 min, the solution was gently blown dry with nitrogen. An AFM image of the sample was obtained using the AFM contact mode, a CSC17/AI BS/50 probe (Agilent Technologies) and a scanning speed of 4.37 cm/s.

### Infrared spectrometric determination

To observe changes in protein molecular structure, a solution of Ara h 1 (0.1 mg/mL) was analysed using Fourier transform infrared spectroscopy (Vertex 70V; Vertex, Boston, MA, USA). A mass of 200 mg KBr (Sinopharm Chemical Reagent Co., Ltd., Shanghai, PR China) was ground in an agate mortar and mixed with 200 μL Ara h 1 (0.2 mg/mL) solution, then transferred to tablet mould and pressed with the pressure increased to 5.1·10^7^ Pa then dropped to zero, after which the sample wafer was inserted into the infrared spectrometer. The infrared spectrum was detected in the range from 4000 to 400 cm^-1^.

### Ultraviolet absorption spectrum

To observe the absorption spectra of Ara h 1 before and after enzymatic hydrolysis, an Ara h 1 (0.1 mg/mL) solution was scanned in the range of 240–450 nm with scanning speed 100 nm/min using UV-Vis spectrophotometer (UV759CRT; Yoke Instrument Co., Ltd., Shanghai, PR China).

### Fluorescence spectrum and determination of hydrophobicity

To observe the changes in the internal structure of proteins by detecting surface hydrophobicity, a volume of 20 μL 80 μM 4,4'-dianilino-1,1'-binaphthyl-5,5'-disulfonic acid dipotassium salt (Bis-ANS, Aladdin, Shanghai, PR China) was added to Ara h 1 (0.2 mg/mL) solution, mixed and transferred to a fluorescence cuvette for analysis with a fluorescence spectrometer (FS F-2500; Shimadzu, Kyoto, Japan). The fluorescence intensity was detected at 400–600 nm by scanning at 25 °C with an excitation wavelength of 350 nm. The scanning conditions were 1200 nm/min, 0.5 s, 500 V and 5 nm.

Different concentrations of Ara h 1 (0.025, 0.05, 0.1, 0.2 and 0.4 mg/mL) were prepared and mixed with 80 μM Bis-ANS, then transferred to a fluorescence spectrophotometer (FS F-2500; Shimadzu) for analysis. The excitation wavelength was 350 nm and the fluorescence intensity was detected at 400–600 nm. The maximum fluorescence intensity was plotted *versus* protein concentration and the curve was fitted by least square method. The slope represents the surface hydrophobicity of the protein (*20*). Each experiment was performed at least three times.

### Circular dichroism

Ara h 1 (0.1 mg/mL) solution was analysed by circular dichroism spectropolarimeter (J-815 CD; JASCO, Kyoto, Japan) to detect its secondary structure. The scanning range was 190–240 nm, the scanning rate was 100 nm/min, the optical diameter was 0.1 cm, the spectral interval was 0.1 nm and the bandwidth was 0.1 nm. The samples were tested 5 times in parallel. The data were analysed using DichroWeb (http://dichroweb.cryst.bbk.ac.uk/html/home.shtml) (*21*) and the secondary structure content was calculated.

### LC-MS/MS spectrometry

Untreated Ara h 1 solution (0.1 mg/mL) and treated with a combination of Flavourzyme and alkaline protease solution were put into a sterilised centrifuge tube and sent to Sangon Biotechnology Co., Ltd (Shanghai, PR China) for LC-MS/MS identification. The differences in protein peptide segments before and after enzymatic hydrolysis were analysed.

### Determination of binding ability of protein to antibody by Western blot

The binding ability of anti-Ara h 1 before and after enzymatic hydrolysis was tested as described by Wang *et al.* (*2*). The protein was separated using a PAGE Gel Fast Preparation Kit (Epizyme). The protein on the gel was transferred to a nitrocellulose membrane and polyvinylidene difluoride (PVDF, Immobilon® transfer membrane, 0.2 μm; Merck Millipore, Burlington, MA, USA) using a sandwich system. After blocking, incubating primary and secondary antibodies, the immunoreactive bands were detected by Chemistar^TM^ high-sig ECL Western blotting substrate (Tanon, Shanghai, PR China).

### Determination of binding ability of protein to antibody by ELISA

The binding ability of Ara h 1 to antibody was determined by indirect ELISA. Ara h 1 (10 μg/mL, 200 μL/well) was placed on the plate labelled with horseradish peroxidase (HRP). The samples were coated at 4 °C overnight. A volume of 200 μL skimmed milk solution (5 %) was added to each well, the plate was sealed at 37 °C for 1 h and then washed three times with phosphate buffer containing Tween 20 (PBST). The plate was first incubated at 37 °C for 2 h with mouse anti-Ara h 1 monoclonal antibodies (1:400 dilution) and washed with PBST five times. Then, it was incubated at 37 °C for 1 h with goat anti-mouse HRP-bound anti-IgG antibodies (1:2000 dilution) and washed with PBST 5 times. Finally, tetramethylbenzidine solution (Tanon) was added for colour developing for 10-15 min. The absorbance was measured at 450 nm with a single function light absorption protease plate analyser (EMax Plus; Molecular Devices, San Jose, CA, USA).

### Culture of Caco-2 cells

The Caco-2 cells were cultured at 37 °C and 5 % CO_2_ as described by Wang *et al.* (*2*). The human colorectal cancer cell line Caco-2 (Zhong Qiao Xin Zhou Biotechnology Co., Ltd, Shanghai, PR China) was cultured with RPMI cell culture medium (Gibco, Thermo Fisher Scientific, San Jose, CA, USA), 10 % foetal bovine serum (Gibco) and 1 % penicillin-streptomycin solution (Aibo, Hangzhou, PR China). The cells were cultured for 2-3 days to achieve fusion, and to assist in the process of cell growth and differentiation, fresh culture medium was added daily. The growing cells were digested with trypsin and passaged continuously at a ratio of 1:2.

### Detection of Caco-2 cytokines by RT-PCR

RNA extraction and real-time polymerase chain reaction (RT-PCR) steps in Caco-2 cells were consistent with those previously described by Wang *et al.* (*2*). RT-PCR was performed using the real time PCR detection system (CFX96; BioRad, Shanghai, PR China). The total cDNA was standardised with glyceraldehyde-3-phosphate dehydrogenase (GAPDH) as the housekeeper gene. The gene primers used in this paper are as follows: IL-5 forward: GCTTCTGCATTTGAGTTTGCTAGCT and reverse: TGGCCGTCAATGTATTTCTTTATTAAG, IL-6 forward: TCCATCCAGTTGCCTTCTTG and reverse: AAGCCTCCGACTTGTGAAGTG, and GAPDH forward: CCACCCATGGCAAATTCC and reverse: TGGGATTTCCATTGATGACCAG.

### Detection of Caco-2 cytokines

We determined IL-5 and IL-6 concentrations in Caco-2 cell supernatants after 24 h of culture with ELISA kits (Lianke, Hangzhou, PR China) according to the manufacturer’s instructions.

### Statistical analysis

The data are expressed as the mean value±S.D. and all assays from each independent treatment were carried out in triplicate. Appropriate data were analysed to determine statistical significance using ANOVA (*22*). The *t*-test and Fisher’s least significant difference (LSD) test were used to examine differences between group means.

## RESULTS AND DISCUSSION

### Effect of protease treatment on the binding ability of Ara h 1 to antibodies

**Fig. 1:**
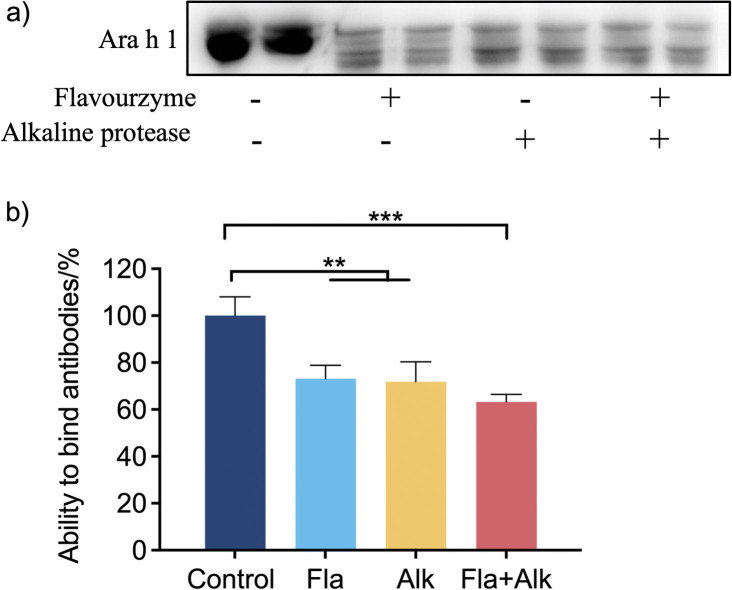
Effect of protease treatment on the *in vitro* sensitization of Ara h 1: a) Western blot test of the binding ability of Ara h 1 and antibody, b) indirect ELISA test of the binding ability of Ara h 1 and antibody. Control=Ara h 1 without protease treatment, Fla=Ara h 1 after the treatment with Flavourzyme, Alk=Ara h 1 after the treatment with alkaline protease, Fla+Alk=Ara h 1 after the treatment with Flavourzyme and alkaline protease. Data are expressed as mean±S.D. of three independent experiments. *p<0.05, **p<0.01, ***p<0.001

*In vitro* tests for detecting allergens commonly include serum-specific antibody assays and cell model allergenicity evaluations (*2*, *23*-*25*). To detect the antigenicity and potential allergenicity of Ara h 1 after enzymatic hydrolysis, Western blot and ELISA were used to conduct *in vitro* antigen-antibody binding experiment with mouse anti-Ara h 1 monoclonal antibody (Fig. 1a). The band of Ara h 1 became shallow after protease treatment, indicating that the binding ability of Ara h 1 to antibody decreased. This phenomenon was verified using indirect ELISA (Fig. 1b). Compared to the control group, the binding ability slightly decreased after the treatment with single protease, while it decreased significantly, by 37.7 %, after the combined treatment with Flavourzyme and alkaline protease. Hence, a significant decrease in the binding ability after protease treatment can reduce the *in vitro* allergenicity of Ara h 1. Similar to the results of the present study, Yang *et al.* (*26*) treated egg white with human alkaline protease. The ability of the hydrolysed protein to bind to IgG and IgE was determined by competitive ELISA using rabbit polyclonal antibodies and serum from patients with egg allergy. Their study showed that the hydrolysis of protein by alkaline protease helps to reduce the binding of IgG and IgE in the hydrolysate, thus reducing the sensitization of the protein. Mikiashvili *et al*. (*27*) treated peanut allergens Ara h 1, 2, 3 and 6 with Alcalase and papain, they found that enzyme treatment effectively reduced overall IgE-binding of peanuts.

### Effects of protease treated Ara h 1 on Caco-2 cell cytokine expression and secretion

**Fig. 2:**
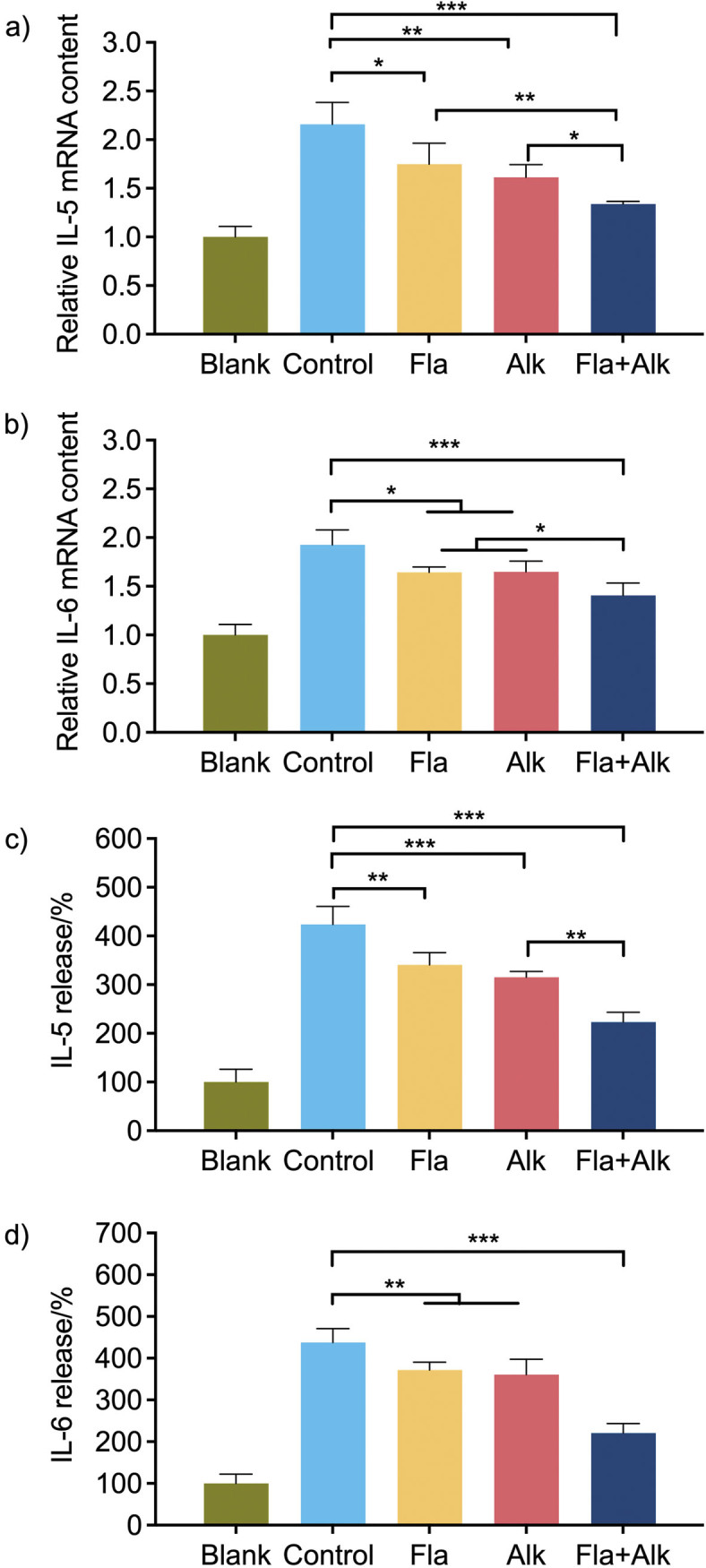
Effects of the gene expression of: a) IL-5, b) IL-6, and the secretion level of inflammatory factors of: c) IL-5 and d) IL-6 on Caco-2 after protease treatment. Blank=without Ara h 1, control=Ara h 1 without protease treatment, Fla=Ara h 1 after the treatment with Flavourzyme, Alk=Ara h 1 after the treatment with alkaline protease, Fla+Alk=Ara h 1 after the treatment with Flavourzyme and alkaline protease. Data are expressed as mean±S.D. of three independent experiments. *p<0.05, **p<0.01, ***p<0.001

In order to study the expression of Ara h 1 proinflammatory factor, Caco-2 cells were incubated with the peanut allergen Ara h 1, which was treated using different methods (Flavourzyme, alkaline protease, or both) for 24 h. The gene expression of IL-5 and IL-6 in Caco-2 cells was determined by RT-PCR (Fig. 2a and Fig. 2b). Compared to the blank group, which was not treated with peanut protein Ara h 1 in Caco-2 cells, the expression of cytokines IL-5 and IL-6 in Caco-2 cells was upregulated after Ara h 1 treatment. This indicated that Ara h 1 promoted inflammation and allergic reaction in Caco-2 cells. Because the originally upregulated IL-5 and IL-6 genes were suppressed after protease treatment, the proinflammatory contents of Ara h 1 were effectively reduced. The secretion of the inflammatory cytokines IL-5 and IL-6 was evaluated by ELISA, and the secretion of IL-5 and IL-6 decreased significantly in protease-treated Ara h 1 group (Fig. 2c and Fig. 2d). These results indicate that the protease treatment effectively reduced the proinflammatory ability of Ara h 1.

Ara h 1 is immunogenic and can be taken up by cells to trigger allergic reactions, resulting in the release of inflammatory factors such as IL-5 (*28*). Tian *et al*. (*29*) emphasised the effects of simulated gastric juice digestion on the immunoreactivity and proinflammatory properties of recombinant Ara h 1. Their results showed that Ara h 1 led to increased cytokine secretion and inflammation through activation of the NF-κB pathway. Deng *at al.* (*30*) and Lin *et al.* (*31*) found that ovalbumin induced the increase of IL-5 in the spleen and intestine of mice. The ovalbumin food allergy model of Harusato *et al.* (*32*) showed that the expression of IL-5 cytokines in the colon of mice increased after food allergy. After subjecting Ara h 1 to enzymatic cleavage, we observed a decrease in the expression and secretion amounts of the proinflammatory cytokines IL-5 and IL-6. This indicated a reduction in the proinflammatory capability of Ara h 1. Compared to the single enzyme treatment, the expression and secretion levels of proinflammatory factors in the Flavorzyme and alkaline protease treatment groups were significantly decreased. This may be due to protease treatment (especially the treatment with two enzymes), which disrupted the antibody-binding epitopes and reduced the immunogenicity of Ara h 1. Whether protease treatment affects the expression of other proinflammatory factors requires further research.

### Effects of protease treatment on particle size and aggregation of Ara h 1

**Fig. 3:**
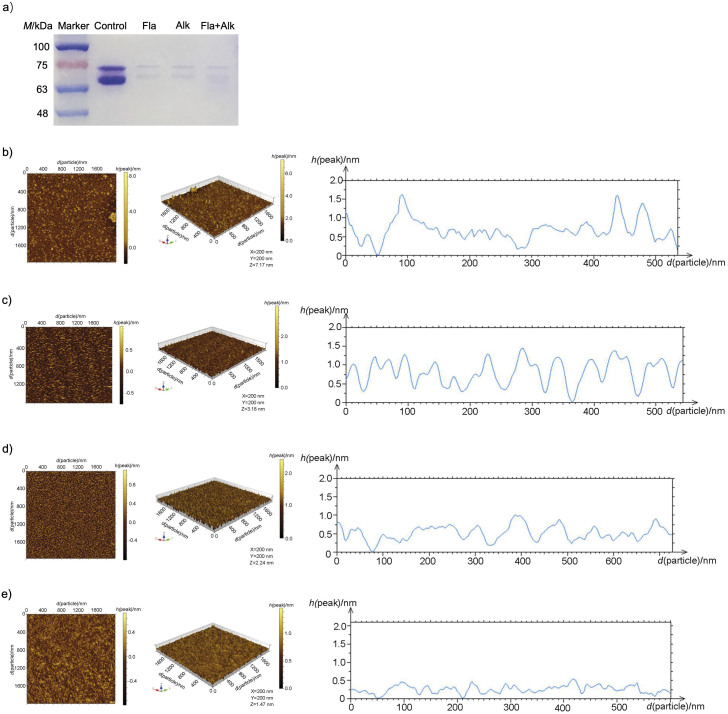
Effect of protease treatment on the electrophoretic bands and particle aggregation of Ara h 1: a) electrophoretogram, and atomic force microscope observation of: b) untreated Ara h 1, c) treated with Flavourzyme (Fla), d) treated with alkaline protease (Alk), and e) treated with Flavourzyme and alkaline protease (Fla+Alk)

To investigate the relationship between the reduced allergenicity and the protein structure of Ara h 1, the secondary and tertiary structures of the Ara h 1 protein (before and after protease treatment) were analysed by SDS-PAGE and atomic force microscopy. Purified Ara h 1 was subjected to enzymolysis as described above and then electrophoresed. Fig. 3a shows that the protein was degraded after enzymolysis. A combined treatment with Flavourzyme and alkaline protease had a better effect on protein degradation than single protease. Atomic force microscopy was used to further analyse the effect of enzymolysis on the degree of aggregation and particle size of Ara h 1. Protein aggregation affects the potential allergenicity of Ara h 1 (*33*). Fig. 3b, Fig. 3c, Fig. 3d and Fig. 3e show that the average particle size of Ara h 1 and the peak height of protein decreased after enzymatic hydrolysis, which indicates that the protease treatment can reduce the peak height of protein and make the protein more dispersed, especially in the treatment with Flavourzyme and alkaline protease. Our study showed that the structure of Ara h 1 was greatly changed after protease treatment, the protein was degraded and the particle size and aggregation degree were reduced.

### Effect of protease treatment on the secondary and tertiary structure of Ara h 1

**Fig. 4:**
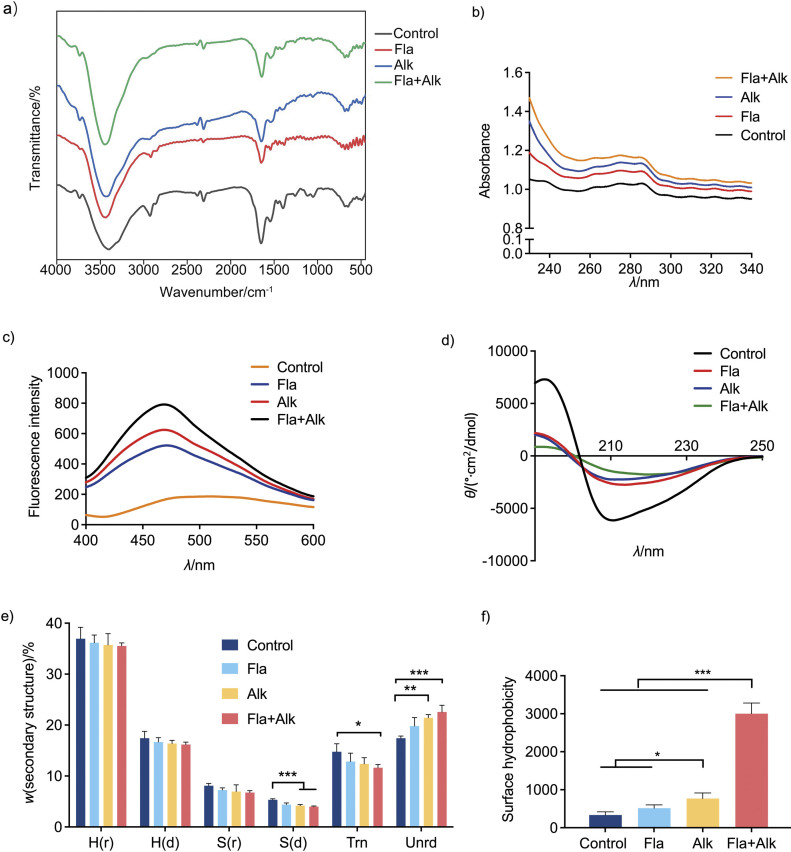
Effect of protease treatment on the structure of Ara h 1: a) infrared spectrogram, b) ultraviolet spectrum, c) fluorescence spectrogram, d) circular dichroism analysis, e) analysis of secondary structure content, and f) surface hydrophobicity. Control=Ara h 1 without protease treatment, Fla=Ara h 1 after treatment with Flavourzyme, Alk=Ara h 1 after treatment with alkaline protease, Fla+Alk=Ara h 1 after treatment with Flavourzyme and alkaline protease. H(r) and H(d)=α-helix, S(r) and S(d)=β-strand, Tm=β-turn, Unrd=random coil. Data are expressed as mean±S.D. of three independent experiments. *p<0.05, **p<0.01, ***p<0.001

The infrared spectra of Ara h 1 before and after enzymatic hydrolysis are shown in Fig. 4a. We can observe the changes of groups in the structure of Ara h 1, which shows that the peak positions of Ara h 1 had little influence after the enzymatic treatment. The UV absorption spectrum of proteins mainly depends on the presence of side chain groups containing residues such as Tyr and Trp (*34*). Therefore, a large number of amino acid residues are produced after enzymatic hydrolysis of proteins, especially Tyr and Trp residues. In their natural state, most hydrophobic amino acid residues constituting the protein are located in the interior, forming a hydrophobic core that is crucial for maintaining the compact three-dimensional structure of the protein. In order to further observe the structural changes of Ara h 1, we used the UV detection of spectra and the results are shown in Fig. 4b. Compared with the control group, the UV absorption of peanut protein after enzymatic hydrolysis was increased, especially after Flavourzyme and alkaline protease treatment. This finding also agrees with the result reported by Zhang *et al.* (*35*) and Mei *et al.* (*36*). Fig. 4c shows the fluorescence spectrum of Ara h 1 after protease treatment, with the emission peak at 470 nm. The peak intensity was the highest after the combined treatment with Flavourzyme and alkaline protease, indicating that the amino acid residues increased and the protein structure changed significantly after the treatment, resulting in an increase in the binding ability of the protein to the fluorescent probe. These results indicated that the protease treatment changed the spatial structure of the protein, resulting in the exposure of hydrophobic groups originally located inside the protein, especially aromatic amino acids containing conjugated double bonds.

Ara h 1 is a trimeric complex and its monomers have a typical cupin structure. Most IgE-binding epitopes (*e.g.* 11-13 and 18-22) of Ara h 1 overlap in the α-helix region of the C-terminal of the cupin structure, and its allergenicity may be related to the stability of the cupin structure. The protein was characterised by circular dichroism (Fig. 4d and Fig. 4e). The effect of protease treatment on the secondary structure of the proteins was analysed using circular dichroism. Fig. 4d shows that the left and right rotational absorbance of the proteins after protease treatment decreased significantly. Random coils increased, while the other secondary structures showed a trend of decrease (Fig. 4e) and, among them, regular β-turn decreased the most. Among the three treatments, the Flavourzyme and alkaline protease treatment had the greatest influence on protein secondary structure. After enzymolysis, the content of secondary structures decreased overall, and the regular β-turn decreased greatly, indicating that enzymatic hydrolysis disrupted the original arrangement of the protein and made its structure more unstable and quite disordered. As the change in secondary and tertiary structures also leads to the destruction of conformational epitopes, the binding ability to antibodies and allergenicity also decreased. Compared to protease treatment alone, the combined Flavourzyme and alkaline protease treatment had a more significant effect on the allergenicity and protein structure. Previous studies have also shown that the secondary structure of the protein changed after enzyme treatment; Park *et al*. (*37*) treated tropomyosin with transglutaminase and tyrosinase, its structure was changed, α-helices decreased by 20.1 and 15.2 % and β-turn increased by 5.8 and 6.2 %.

The change in surface hydrophobicity was also measured (Fig. 4f) and the results shown that the hydrophobicity of Ara h 1 increased after the protease treatment, indicating that the conformational structure of the protein was destroyed and the hydrophobic groups inside the protein were exposed to the surface. Notably, the Flavourzyme and alkaline protease treatment had the greatest effect on the hydrophobicity of the protein surface, which was 8.95-fold higher than the untreated Ara h 1. Different from our results, Ahmed *et al*. (*38*) found that the surface hydrophobicity decreased when using laccase/caffeic acid and transglutaminase to alleviate shrimp tropomyosin, which might be due to the aggregation of protein by hydrophobic interactions, creation of hydrophilic groups (containing −NH_2_ and −OH) and partial unfolding of protein.

### LC-MS/MS analysis of protein structure and allergenicity of untreated and protease-treated Ara h 1

**Fig. 5:**
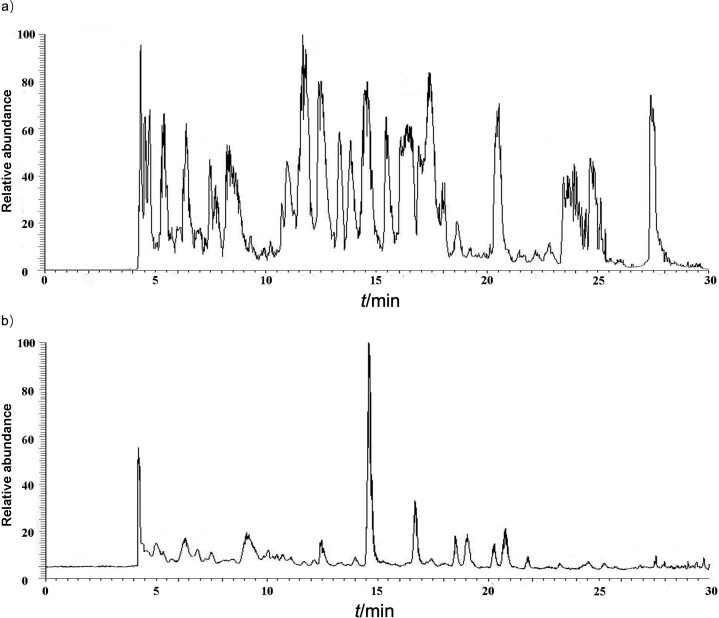
LC-MS/MS analysis of Ara h 1 protease treatment: a) untreated Ara h 1, and b) Ara h 1 treated with Flavourzyme and alkaline protease

Because of the significant influence of Flavourzyme and alkaline protease treatment on protein structure and allergenicity, the site of enzyme treatment, Ara h 1 (untreated and after the treatment with Flavourzyme and alkaline protease) was investigated further using LC-MS/MS (Fig. 5). As a result, 14 different peptides were found in Ara h 1 (untreated and treated), and these sites were mainly hydrolysed by Flavourzyme and alkaline protease, resulting in conformational changes in the protein. In the work of Tian *et al.* (*39*), the epitopes located in Ara h 1 393-402 and Ara h 1 498-508 were recognized in 60 and 90 %, respectively, of Chinese patients with peanut allergy. Hu *et al*. (*40*) also found that 27 integral sequential epitopes were identified in newly produced peptides after Flavourzyme proteolysis of β-conglycinin. In our study, both linear epitopes were effectively destroyed by protease treatment. These results indicate that protease treatment of the secondary structure of the protein destroys its original conformational structure and may affect the allergenicity of Ara h 1. This protein was identified as the main peanut allergen. After enzymolysis, the 14 obtained peptides differed from the untreated ones (Table 1). Except for peptide 1, the other 13 peptides were located in the core region of Ara h 1. It can be speculated that Flavourzyme and alkaline protease changed the spatial structure of Ara h 1, which might affect its allergenicity by the enzymolysis of these main fragments.

**Table 1:** The sequence of degraded protein peptides after the treatment with Flavourzyme and alkaline protease

Number	Start site	Stop site	Sequence
1	153	166	EGEQEWGTPGSHVR
2	167	180	EETSRNNPFYFPSR
3	205	213	QFQNLQNHR
4	255	265	SFNLDEGHALR
5	357	370	SSENNEGVIVKVSK
6	367	378	VSKEHVQELTK
7	386	401	GSEEEDITNPINLR
8	402	403	EGEPDLSNNFGK
9	414	421	LFEVKPDK
10	422	434	KNPQLQDLDMMLT
11	440	451	EGALMLPHFNSK
12	461	472	GTGNLELVAVRK
13	504	514	LKEGDVFIMPAAH
14	578	588	ESHFVSARPQ

These results provide a theoretical basis for reducing peanut allergenicity. The mechanism through which enzymatic hydrolysis reduces allergenicity involves protein uptake and transport within cells. Additionally, understanding the pathways associated with inflammatory factors and employing animal models is crucial.

## CONCLUSIONS

This study showed that the binding affinity of Ara h 1 to the antibody was reduced after the treatment with protease. Compared to the untreated Ara h 1 group, the expression and secretion levels of the proinflammatory cytokines IL-5 and IL-6 in Caco-2 cells were significantly reduced after enzymatic digestion, demonstrating that protease treatment effectively reduced the proinflammatory response and potential sensitization caused by Ara h 1. This indicated that the protease reduced the proinflammatory level and potential sensitization of Ara h 1 by degrading the protein particles and changing the content of secondary structures. Our study provides an effective method for reducing peanut allergy to Ara h 1 but its mechanism needs to be studied further to provide a theoretical basis for the development of low-allergenic peanut-based products.
